# New Polyprenylated Phloroglucinol and Other Compounds Isolated from the Fruits of *Clusia nemorosa* (Clusiaceae)

**DOI:** 10.3390/molecules200814326

**Published:** 2015-08-06

**Authors:** Rafaela Oliveira Ferreira, Tania Maria Sarmento da Silva, Mário Geraldo de Carvalho

**Affiliations:** 1 Departamento de Química, Universidade Federal Rural do Rio de Janeiro, 23890-000 Seropédica, Brazil; E-Mail: mgeraldo@ufrrj.br; 2 Departamento de Ciências Moleculares, Universidade Federal Rural de Pernambuco, 52171-900 Recife, Brazil; E-Mail: sarmentosilva@gmail.com

**Keywords:** Clusiaceae, *Clusia nemorosa*, phloroglucinol, nemorosic acid, flavonoid, triterpenoid

## Abstract

*Clusia nemorosa* has been widely used in folk medicine to treat various ailments, including headaches and inflammation. Investigation of the fruits of *Clusia nemorosa* (Clusiaceae) led to the isolation and characterization of a new phloroglucinol derivative, named 6*S*,8*S*,28*S*-nemorosic acid (**1**), together with seven known compounds: friedelin (**2**), β-sitosterol (**3**), stigmasterol (**4**), β-sitosterol glycoside (**5**), kaempferol (**6**), quercetin (**7**) and dimethyl citrate (**8**). The structures were determined by extensive 1D- and 2D-NMR, CD and MS spectroscopic analyses.

## 1. Introduction

The Clusiaceae family, also known Guttiferae, is characterized by the presence of flower resin and latex in most of its species [1]. The genus *Clusia* presents a notable metabolite diversity, including benzophenones, xanthones and biflavonoids [2,3,4,5].Several species are used in folk medicine all around the world to treat rheumatism, stomach problems and as a purgative [6]. Phytochemical and pharmacological studies of plants belonging to this genus have reported many biological activities, such as antibacterial, antioxidant, antitumour, antimicrobial and anti-inflammatory properties [7]. (–)-Nemorosonol, a prenylated benzophenone, exhibited antimicrobial activity against *Escherichia coli*, *Staphylococcus aureus*, *Bacillus subtilis*, *Micrococcus luteus*, *Aspergillus niger*, *Trichophyton mentagrophytes* and *Candida albicans* [8]. The biflavonoids GB1-7′′*O*-glucoside and GB1a-7′′*O*-glucoside showed promising free radical scavenging capacity in *in vitro* assays [5]. Phloroglucinol derivatives exhibited chemopreventive properties and inhibited NO production [9]. The *Clusia* genus thus represents an important source of bioactive compounds.

*Clusia* *nemorosa* Mey., popularly known as “pororoca”, is a tree that is widespread in the northeast region of Brazil [10]. This species has been widely used in folk medicine to treat headaches and inflammation [11]. Prior phytochemical studies of the fruits of this species led to the isolation of a polyisoprenylated benzophenone and phloroglucinol derivatives [12,13]. The additional study of this species presented herein allowed us to identify a new phloroglucinol derivative, named nemorosic acid (**1**), as well as seven known compounds that were identified for the first time in the fruits: friedelin (**2**), β-sitosterol (**3**), stigmasterol (**4**), β-sitosterol glycoside (**5**), kaempferol (**6**), quercetin (**7**) and dimethyl citrate (**8**).

## 2. Results and Discussion

The fruit of *Clusia nemorosa* was extracted with dichloromethane (CH_2_Cl_2_) and methanol (MeOH). The fractionation of these extracts and the analysis of the fractions allowed us to obtain seven known compounds—friedelin [14], β-sitosterol [15], stigmasterol [15], β-sitosterol glycoside [16], kaempferol [17], quercetin [17] and dimethyl citrate [18]—as well as a new phloroglucinol derivative, named nemorosic acid (**1**).

**Figure 1:**
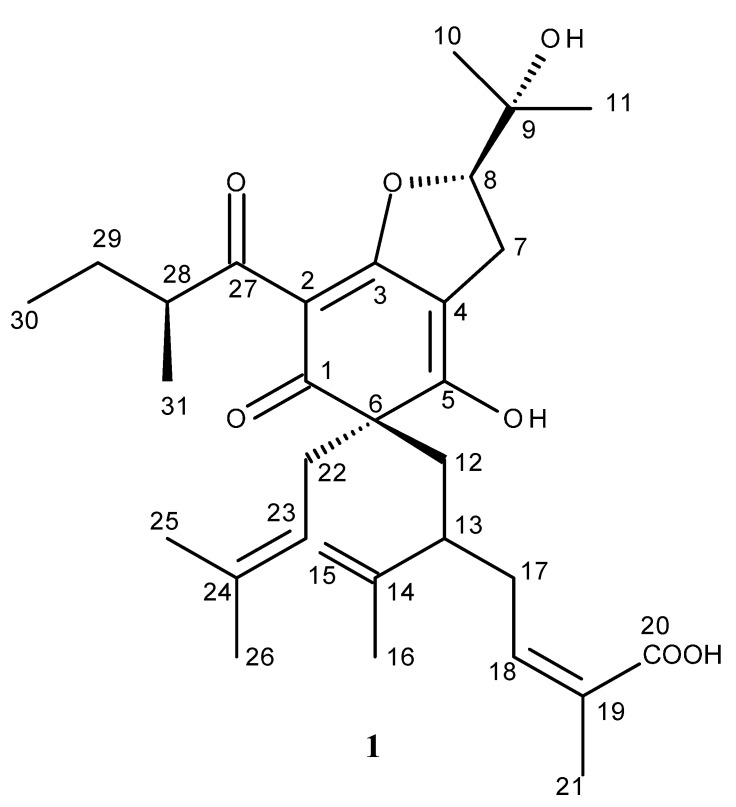
Structure of compound **1**.

Nemorosic acid (**1**) was isolated as an optically active ([αD] = −5.34°) yellow gum. The molecular formula was established as C_31_H_44_O_7_ on the basis of negative ESI-MS data (found *m*/*z* 527.3220, [M − H]^−^, calc. 527.3009). The IR spectrum displayed absorptions of hydroxyl (3435 cm^−1^) and carbonyl groups (1707 cm^−1^), as well as other signals. The ^1^H- and ^13^C-NMR data (Table 1) suggested that **1** was a phloroglucinol derivative. The structure was defined by the detailed analysis of 2D-NMR (^1^H-^1^H COSY, HMQC, NOESY and HMBC) spectra data and comparison with the data of the known compounds nemorosinic acid B [13], adlupulone [19], and garcinenone F [20]. Comparison of the ^13^C-NMR data of **1** with those of adlupulone, which was isolated from *Humulus lupulus* L. [19], revealed that **1** contained two enolic C-atoms at δ(C) 170.4 (C(3)) and 191.9 (C(5)), one C=O group at δ(C) 205.5 (C(1)), and three quaternary carbons at δ(C) 103.3 (C(2)), 109.6 (C(4)), and 60.9 (C(6)), which was the same as for adlupulone and garcinenone F. Thus, **1** was characterized as having a cyclohexa-2,4-dien-1-one moiety. Extensive analysis of ^1^H- and ^13^C-NMR, together with HMBC spectra, indicated the presence of a 3-methylbut-2-en-1-yl group (δ(H) 2.53 (brd, *J* = 7.3 Hz, 2H, H-22), 4.77 (m, H-23), 1.51 (s, 3H, H-26), and 1.48 (s, 3H, H-25); δ(C) 42.2 (C(22)), 116.8 (C(23)), 135.6 (C(24)), 25.8 (C(26), and 17.8 (C(25)), an oxidized lavandulyl group (δ(H) 2.26 (brd, 10Hz, H-12a), 2.08 (m, H-12b), 2.14 (m, H-13), 4.51/4.42 (brs, H-15a, 15b), 1.48 (s, 3H, H-16), 2.14 (m, H-17), 6.64–6.68 (m, H-18), and 1.77 (s, 3H, H-21); δ(C) 40.4 (C(12)), 44.4 (C(13)), 145.7 (C(14)), 114.1 (C(15)), 17.6 (C(16)), 33.3 (C(17)), 142.5 (C(18)), 127.5 (C(19)), 172.2 (C(20)), and 12.0 (C(21)) (Figure 1, Table 1), a 2-methylbutanoyl group (δ(H) 3.53 (sextet, 1H, H-28), 1.45 (m, H-29a, 1.75 (m, H-29b), 0.93 (t, 7.3 Hz, 3H, H-30), and 1.18 (d, 6.8Hz, 3H, H-31); δ(C) 196.5 (C(27)), 40.7 (C(28)), 27.4 (C(29)), 11.9 (C(30)), and 17.2 (C(31)), and a 2,3-dioxy-3-methylbutyl moiety (δ(H) 2.87–3.01 (m,H-7a,7b), 4.75–4.83 (m, 1H, H-8), 1.32 (s, 3H, H-10), and 1.26 (s, 3H, H-11)); δ(C) 26.6 (C(7)), 92.4 (C(8)), 71.8 (C(9)), 24.8 (C(10)), and 24.6 (C(11)) (Table 1). The positions of the substituents were deduced by analysis of the HMBC data (Table 1, Figure 1). The HMBC cross-peaks of CH_2_(22) and CH_2_(12) with δ(C) 205.5 (C(1), 191.9 (C(5), and 60.9 (C(6), established that a 3-methylbut-2-en-1-yl and lavandulyl group were linked to C(6). The observed signals on the HMBC spectrum of ^n^*J*_HC_ of CH_2_(7) with δ(C) 109.3 (C(4)), 170.4 (C(3)), 92.4 (C(8)), and 71.8 (C(9)) allowed to located the 2,3-dihydro-2-(1-hydroxyl-1-methylethyl)-furan moiety at C(3) and C(4), different from those of garcinenone F [20]. Besides other long range coupling signals, the value 191.9 of **1** (with H-22 and H-17) allowed to locate the enolic OH group at C(5), different of C(5) in garcinenone F [20]. The remaining 2-methylbutanoyl group was attached at C(2) based on the ^3^*J*_HC_ of H-28 and C(2) (δ_C_ 103.5) (Table 1). The chemical shifts of H_a_ and H_b_ of C(7), C(17), and C(22) were overlapped in the ^1^H-NMR (see supplementary material).

**Table 1:** ^1^H (500 MHz) and ^13^C (125 MHz) NMR data of **1** in CDCl_3_.

Position	δ_H_	δ_C_	HMBC(^2,3^*J*_H-C_)
1	-	205.6	-
2	-	103.5	-
3	-	170.4	-
4	-	109.3	-
5	-	191.9	-
6	-	60.9	-
7	2.87–3.01 (m)	26.6	C-3; C-4; C-5; C-8; C-9
8	4.75–4.83 (m)	92.4	C-3; C-4 ;C-10; C-11
9	-	71.8	-
10	1.32 (s)	24.8	C-8; C-9; C-11
11	1.26 (s)	24.6	C-8; C-9; C-10
12	2.26 (br d, 10 Hz, H-12a); 2.08 (m, H-12b) ^a^	40.4	C-1; C-5; C-6; C-13; C-14; C-17
13	2.14 (m)	44.4	C-6; C-14; C-15; C-17
14	-	145.7	-
15	4.51 (brs, H-15a); 4.52 (brs, H-15b) ^a^	114.1	C-13; C-14; C-16
16	1.48 (s)	17.8	C-13; C-14; C-15
17	2.14 (m)	33.3	C-12; C-14; C-18; C-19
18	6.68 (brt, 6.6 Hz)	142.5	C-13; C-17; C-19; C-20; C-21
19	-	127.5	-
20	-	172.2	-
21	1.77 (s)	12.0	C-18; C-19; C-20
22	2.53 (brd, 7.3 Hz)	42.2	C-1; C-5; C-6; C-12; C-23; C-24
23	4.75–4.83 (m)	116.8	C-22; C-25; C-26
24	-	135.6	-
25	1.48 (s)	17.6	C-23; C-24;C-26
26	1.51 (s)	25.7	C-23; C-24; C-25
27	-	196.5	-
28	3.53 (sextet, 6.8 Hz)	40.7	C-2;C-27; C-29; C-30; C-31
29	1.45–1.50 (m, H-29a); 1.75 (m, 29b) ^a^	27.4	C-27; C-28; C-30; C-31
30	0.93 (t, 7.3 Hz)	11.9	C-28; C-29
31	1.18 (d, 6.8 Hz)	17.2	C-27; C-28; C-29

^a^ Correlation from ^1^Hx^1^H-COSY and HMQC spectra.

**Figure 2:**
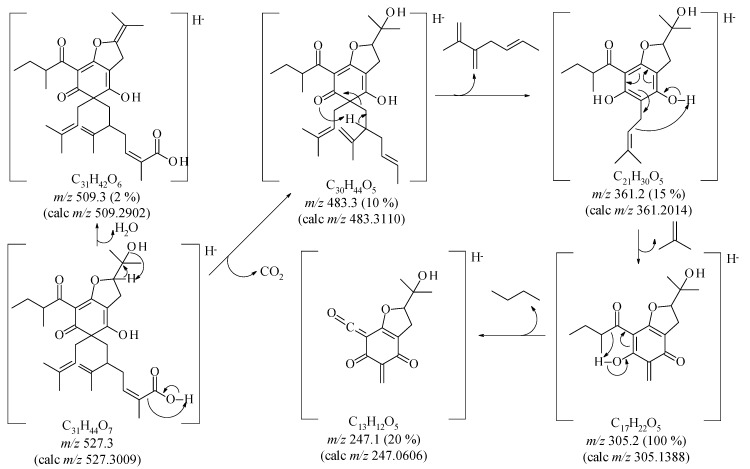
Proposed fragmentation to justify the principal peaks detected in the mass spectrum.

**Figure 3:**
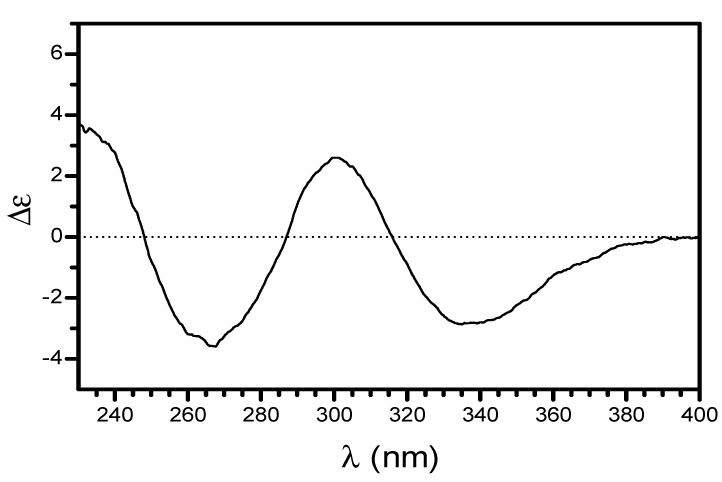
Circular dichroism spectrum of **1** in methanol.

As described by Monache *et al*. [13], there is a possibility of epimers at C(28), besides the keto-enolic equilibrium to form tautomeric structures in these kinds of compound. In the case of **1**, in addition there exists the possibility of epimers at C(8), and different conformers of the linked groups at C(6). These structural properties justify the additional signals in the ^1^H- and ^13^C-NMR spectra. Moreover the proposed structure **1** is the major component which provided the chiral properties discussed below. The detailed MS analysis (Figure 2) was used to confirm the structure of **1**. Various polyisoprenylated phloroglucinol derivatives have being isolated from genus *Clusia* [13]. Most of them belong to a bicyclononane ring system, as in the case of nemorosonol [12], whereas nemorosic acid, having a cyclohexa-2,4-dien-1-one moiety, is similar to nemorosinic acid B, isolated from *Clusia nemorosa* [13], lupulone, which occur in *Humulus lupulus* [9], and garcinenone F, isolated from *Garcinia xanthochymus* [20]. Lupulone derivatives possess one or more stereogenic centres, but these compounds were racemic ([α]_*D*_ = 0) [9]. However, **1** was optically active ([α]_*D*_ = −5.34°), and the CD curve is presented in Figure 3. Therefore, the configuration of **1** was proposed as 6*S*,*8S*,*28S-* according to the negative and positive Cotton effect at 340 and 300 nm, respectively (Figure 3), such as (−)-nemorosonol [8] and marmesinin [20], with negative and positive Cotton effect at 250 and 230 nm, containing identical furan system with *S* configuration [21]. Therefore **1** is a new phloroglucinol derivative whose structure was defined as (–)-6*S*,*8S*,*28S*-6-(lavundolyl-6-carboxy)-6-(3-methylbut-2-en-1-yl)-2-(2-methylbutanoyl)-[2′-hydroxyiso-propyl-2′,3′-dihydrobenzofurane-(2′,3′:3,4)]-5-hydroxycyclohexa-2,4-dienone, named as nemorosic acid. The known compounds were identified by 1D- and 2D-NMR spectroscopic data analysis, and comparison with literature data [14,15,16,17,18].

## 3. Experimental Section

### 3.1. General Procedures

The melting points were determined with a Mel-temp II apparatus and were uncorrected. IR spectra were obtained with KBr discs and NaCl film using a Vertex-70 spectrophotometer (Bruker Corporation, Billerica, MA, USA). UV spectra were obtained on a Shimadzu UV-mini 1240 UV-VIS spectrophotometer (Quioto, Kansai, Japan). ^1^H- and ^13^C-NMR experiments were performed on Bruker Avance III 500 (Billerica, MA, USA) (500 for ^1^H and 125 MHz for ^13^C) and Bruker AC-400 (400 for ^1^H and 100 MHz for ^13^C) spectrometers using DMSO-*d*_6_, methanol-*d*_4_, pyridine-*d*_5_ or CDCl_3_ as solvents with TMS as the internal reference. Chemical shifts are in ppm and the *J* values in Hz. Mass spectra were registered on a microTOFq II–ESI–TOF spectrometer (Bruker Corporation, Billerica, MA, USA) using ESI- in negative mode. Optical rotation was measured with a Jasco P-2000 polarimeter (Easton, MO, USA). The circular dichroism spectrum was realized at DC J-180 Jasco PT4235\190–400 nm. Column chromatography was performed using silica gel (Vetec, Duque de Caxias, RJ, BR and Sigma-Aldrich, St. Louis, MO, USA) (0.05–0.20 mm) and Sephadex™ LH-20 (Sigma-Aldrich, St. Louis, MO, USA). Silica gel F254 G (Vetec) was used for preparative TLC; aluminium-backed Sorbent silica gel plates, w/UV 254, were used for analytical TLC, with visualization under UV (254 and 366 nm), with AlCl_3_-EtOH (1%), vanillin and iodine vapour.

### 3.2. Plant Material

The fruits of *Clusia nemorosa* Mey. were collected in the Campus of Universidade Federal Rural de Pernambuco (UFRPE), Recife, Brazil, in January 2012. A voucher (number 51474) is deposited at the herbarium of Vasconcelos Sobrinho (PEUFR), Universidade Federal Rural de Pernambuco.

### 3.3. Extraction and Isolation

The dried fruits (130.0 g) were extracted with dichloromethane (2.0 L), followed by extraction with methanol (2.0 L) at room temperature three times. The two extracts were separately concentrated to give 15.0 g of CH_2_Cl_2_ extract and 27.0 g of MeOH extract. Part of the dichloromethane extract (11.0 g) was subjected to column chromatography on silica gel with dichloromethane, ethyl acetate (EtOAc) and methanol (MeOH) as eluents to give three fractions (A1–A3). The A2 fraction (3.0 g) was subjected to repeated silica gel chromatography using hexane–CH_2_Cl_2_, CH_2_Cl_2_–AcOEt and EtOAc–MeOH to furnish the compounds **1** (35.0 mg) and **5** (65.0 mg). The A1 fraction (5.1 g) was further fractionated through silica gel column chromatography using hexane–EtOAc and EtOAc–MeOH to furnish compounds **2** (20.0 mg) and the mixture of **3** and **4** (10.0 mg). Meanwhile, the dry MeOH extract (20.0 g) was suspended in H_2_O and was partitioned with chloroform (3 × 200 mL), ethyl acetate (3 × 200 mL) and *n-*butanol (3 × 200 mL). The EtOAc fraction (3.5 g) was subjected to Sephadex LH-20 column chromatography and eluted with MeOH to give five fractions. Fraction 4 was rechromatographed on Sephadex LH-20 and eluted with MeOH to give **6** (5.0 mg) and **7** (5.0 mg). Fraction 5 was rechromatographed over a silica gel column using CHCl_3_-MeOH (0%–100% methanol) as eluents to give **8** (35.0 mg).

*Nemorosic acid* (**1**). Yellow gum; [α]_D_ –5.34 (MeOH; *c* 0.16); CD: Δɛ (nm): −2.8 (340), +2.6 (300), −3.2 (270), +3.7 (230); UV (MeOH) λ_max_ (nm): 257 and 327; IR: ν^KBr^ (cm^−1^): 3435, 1707, and 1638, besides other signals; ^1^H-NMR (500 MHz, CDCl_3_) and ^13^C-NMR (125 MHz, CDCl_3_), see Table 1; TOF-MS/MS (negative mode) *m*/*z* (rel. int.): 527.3 (70), 509.3 (2), 483.3 (10), 361.2 (15), 305.2 (100) and 247.1 (20), Figure 2.

## 4. Conclusions

Eight compounds, including the novel compound **1**, were isolated from the fruits of *C. nemorosa.* The discovery of this new compound from the genus *Clusia* provides more spectroscopic data to characterize the components of its isolates, as well as contribute to the understanding of the taxonomy and evolution of the genus *Clusia*.

## Supplementary Materials

Supplementary materials can be accessed at: http://www.mdpi.com/1420-3049/20/08/14326/s1.
