# Supplementary material for: New Polyprenylated Phloroglucinol and Other Compounds Isolated from the Fruits of Clusia nemorosa (Clusiaceae)
Source: Molecules. 2015 Aug 6;20(8):14326–33. doi: 10.3390/molecules200814326 (PMC6332069; doi:10.3390/molecules200814326)
Supplement: Supplementary file 1 [file molecules-20-14326-s001.pdf]

Supplementary Materials

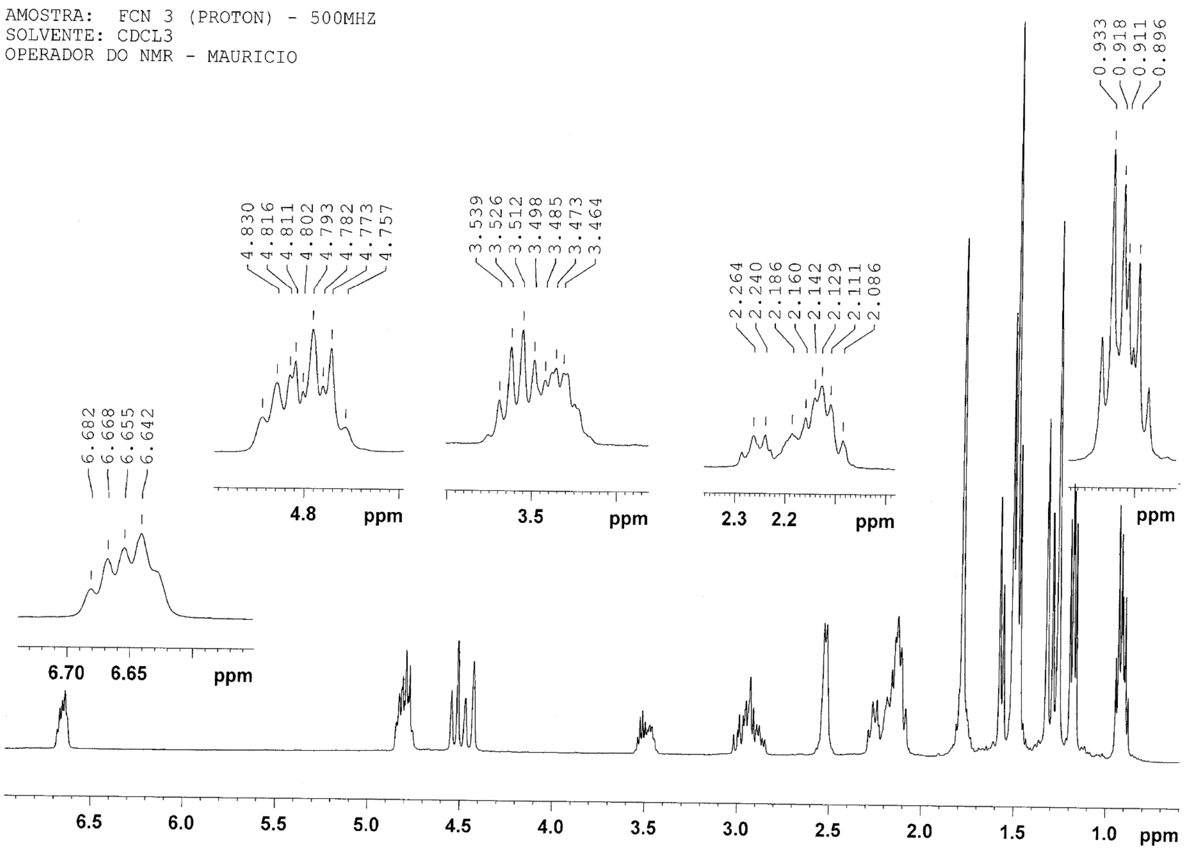

Figure S1. ^{1}H-NMR spectrum of compound **1** at 500 MHz in $CDCl_3$.

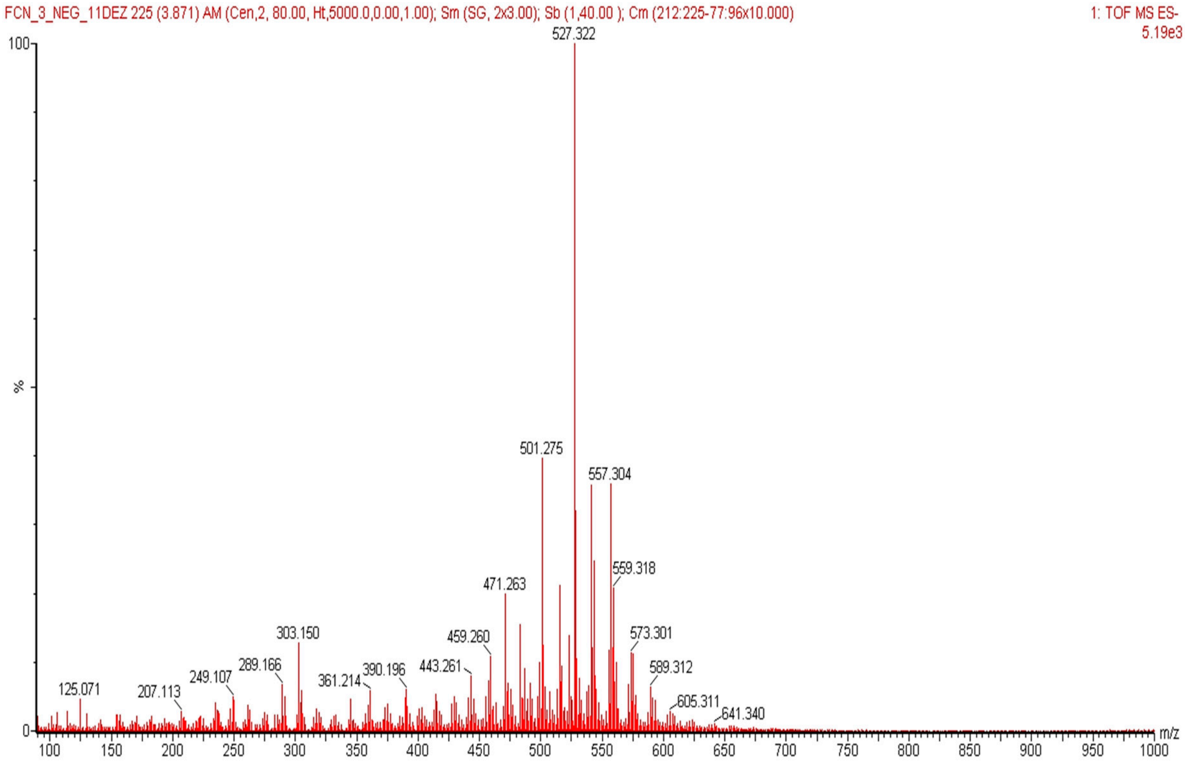

Figure S2. TOF-ESI-MS spectrum of compound **1**.
